# Factors Associated with Anxiety, Depression, and Stress in Peruvian University Students during the COVID-19 Pandemic

**DOI:** 10.3390/ijerph192114591

**Published:** 2022-11-07

**Authors:** Palmer J. Hernández-Yépez, Carlos O. Muñoz-Pino, Valeria Ayala-Laurel, Pavel J. Contreras-Carmona, Fiorella Inga-Berrospi, Víctor J. Vera-Ponce, Virgilo E. Failoc-Rojas, César Johan Pereira-Victorio, Mario J. Valladares-Garrido

**Affiliations:** 1 South American Center for Education and Research in Public Health, Universidad Norbert Wiener, Lima 15046, Peru; 2 Instituto de Investigación en Ciencias Biomédicas, Universidad Ricardo Palma, Lima 15039, Peru; 3 Universidad Tecnológica del Perú, Lima 15046, Peru; 4 Research Unit for Generation and Synthesis Evidence in Health, Universidad San Ignacio de Loyola, Lima 15024, Peru; 5 School of Medicine, Universidad Continental, Lima 15046, Peru; 6 Oficina de Epidemiología, Hospital Regional Lambayeque, Chiclayo 14012, Peru

**Keywords:** anxiety, depression, stress, COVID-19, pandemic

## Abstract

During the COVID-19 pandemic, university students have adopted measures that completely transformed their educational environment, and this has generated an increase in psychological stress. The present study aimed to identify the factors associated with anxiety, depression, and stress in students at a university in Peru during the COVID-19 pandemic in 2020. We conducted a cross-sectional analytical study in students in Lima, Peru. The DASS-21 scale was used to measure levels of depression, anxiety, and stress and associate it with socio-educational and COVID-19-related variables using generalized linear models with Poisson distribution, log link, and robust variance. Of 400 students surveyed, 19.2%, 23.2% and 17.2% of students presented depression, anxiety, and stress, respectively. The frequency of depression (PR = 0.91, 95%CI: 0.84–0.99), anxiety (PR = 0.90, 95%CI: 0.83–0.99) and stress (PR = 0.92, 95%CI: 0.86–0.99) was lower in women. The students of the engineering and business faculty presented a higher frequency of anxiety (PR = 1.11, 95%CI: 1.00–1.22). There was a greater frequency of presenting anxiety, depression and stress in students who worked in a different area of health or did not work. Our results suggest the importance of promoting mental health awareness campaigns in university students due to the constant academic load they have.

## 1. Introduction

The COVID-19 pandemic represents a global health crisis [1]. By September 2020, when this study was conducted, more than 32 million confirmed cases and 990,000 deaths had been reported worldwide [2], while in Latin America the prevalence of COVID-19 had been estimated at 9 million cases and more than 300,000 deaths [3]. The pandemic has highlighted the mental health of the population. Previous studies estimate a 29% prevalence of anxiety due to COVID-19 in the university population [4], while in Latin America a percentage of 33% anxiety has been described [5].

Mental health in the university population is a topic of great interest since university students find themselves in an environment of constant anxiety and stress [6]. The current pandemic has importantly affected the university population, as students have had to adopt measures that completely transform their educational environment. Although previous research has been carried out on the impact on the mental health of university students during the pandemic [7,8], most have been conducted during the first months of the pandemic and have focused on evaluating depression or anxiety and their associated factors [9,10], without considering academic and clinical variables, which our study attempts to evaluate. Additionally, other studies have focused specifically on medical students and have not evaluated the impact of COVID-19 on the mental health of students in other majors [11,12]. Our research is relevant because it considers students from different faculties and delves into sociodemographic, academic, and clinical variables.

For these reasons, there is an urgent need to assess the effects of the pandemic on the mental health and well-being of university students. The present study aimed to identify the factors associated with anxiety, depression, and stress in students at a university in Peru during the COVID-19 pandemic in 2020.

## 2. Materials and Methods

### 2.1. Study Design

We conducted an observational, cross-sectional analytical study during the second academic semester of the year 2020 between the months of September and December at the Universidad Norbert Wiener in Lima, Peru.

### 2.2. Population and Sample

The population consisted of students who attended the 2020-II semester of the faculties of Health Sciences, Law and Political Science, and Engineering and Business of the Universidad Norbert Wiener, which had 6054, 608 and 1563 students, respectively. The sample size was calculated with OpenEpi v.3.01 program applying Fisher’s formula for finite populations, resulting in a sample size of 370 people with a confidence level of 95%. A stratified probabilistic sampling was carried out for each faculty selected for the study. The sample calculated for each faculty was determined in 273, 27 and 70 for the faculties of Health Sciences, Law and Political Sciences and Engineering and Business, respectively.

Students who were enrolled in the 2020-II academic semester and completed the variables of interest were included. Students who did not agree to voluntarily participate in the study were excluded.

### 2.3. Instrument

We used the depression, anxiety, and stress scale (DASS-21). This scale has been validated in Peru for multiple studies in university students [13,14]. The DASS-21 scale consists of 21 items and evaluates the three variables (depression, anxiety, and stress) independently with seven questions for each one using a Likert-type scale with four options: never = 0; sometimes = 1; often = 2; almost always = 3. The results are classified as follows: (a) normal (<5 points), (b) mild (5–6 points), (c) moderate (7–10 points), (d) severe (11–13 points), (e) extremely severe (>14 points). For purposes of this study, the responses were dichotomized into (a) did not present (when the result was normal); (b) it did present (when the result was mild, moderate, severe, and extremely severe).

### 2.4. Variables

The dependent variable was the presence of depression, anxiety, and stress, which was measured operationally with the DASS-21 scale explained above.

The independent variables were the socio-educational data: age (in years), sex (male, female), marital status (single, cohabiting, married, divorced, widowed), relatives with whom they live (accompanied, alone) and having children (no, yes), career (health sciences, engineering, business, law and political science), academic year (general studies and college studies), previous career (no, yes), current type of work (no, related to health, not related to health). Additionally, the students were asked if they had been diagnosed with COVID-19 and if any family member or close friend had been diagnosed with COVID-19.

### 2.5. Study Procedure

The instrument was applied virtually due to social restrictions of COVID-19 pandemic. The survey was applied through Google Forms to the students of the Universidad Norbert Wiener during the second semester of the year 2020. Before the survey was carried out, they were asked to accept an informed consent for their participation in the research. During the entire execution of the study, the autonomy and anonymity of the participants were respected; in the same way, the confidentiality of the results obtained from the applied survey was guaranteed. A database was generated in a spreadsheet in Microsoft Excel program and those records that did not answer the questions with the variables of interest were eliminated from the database, which were 8 records.

### 2.6. Statistical Analysis

All statistical analyses were performed using Stata 15.0. The descriptive analysis was carried out using frequencies and percentages. For the quantitative variable age, mean and standard deviation were reported. We performed a bivariate analysis to estimate the association between depression, anxiety, and stress with sociodemographic, professional, and clinical variables of the students. Subsequently, we carried out a simple and multiple regression analysis to investigate the factors independently associated with depression, anxiety, and stress, using generalized linear models, Poisson distribution family and log link function with robust variance. Prevalence ratios and 95% confidence intervals were estimated.

### 2.7. Ethical Considerations

This study was reviewed and approved by the ethics committee of the Universidad Norbert Wiener, with file No. 078-2020. The surveys were anonymous, and codes were used to maintain confidentiality in the database obtained. Informed consent was given to each research participant.

## 3. Results

Table 1 shows the sociodemographic, academic, and clinical data of the students. The number of students surveyed was 400 students. 58.8% were found to be female and the average age was 24.5 years. More than half (73.0%) reported studying a career belonging to the Faculty of Health Sciences. 24.8% claimed to have been diagnosed with COVID-19. Regarding the DASS-21, 19.2%, 23.2% and 17.2% of students presented depression, anxiety, and stress; respectively.

Table 2 shows the bivariate analysis, where a significant association was found between sex (*p* < 0.001), type of work (*p* < 0.001), and diagnosis of COVID-19 (*p* < 0.001) with the three analyzed variables of the DASS-21 (depression, anxiety, stress). A significant association was also found between the career studied and anxiety and stress (*p* = 002; *p* = 0.016, respectively).

Table 3 shows the simple regression analysis. The frequency of having depression, anxiety and stress was lower in women by 20% (PR = 0.80, 95%CI: 0.73–0.88), 21% (PR = 0.79, 95%CI: 0.71–0.88) and 19% (PR = 0.81, 95%CI: 0.75–0.88); respectively. Likewise, students who belonged to the engineering and business faculty had a higher frequency of having depression, anxiety and stress in 15% (PR = 1.15, 95%CI: 1.04–1.26), 23% (PR = 1.23, 95%CI: 1.11–1.37), and 15% (PR = 1.15, 95%CI: 1.05–1.25), respectively, when compared to health sciences students. In addition, those students who worked in a different health area or did not work, those who were not diagnosed with COVID-19 and those who did not have a relative diagnosed with COVID-19 presented a higher frequency of having depression, anxiety and stress.

Table 4 shows the multiple regression analysis. The association of lower frequency to having depression, anxiety and stress in women was maintained; 9% (PR = 0.91, 95%CI: 0.84–0.99), 10% (PR = 0.90, C95%I: 0.83–0.99), 8% (PR = 0.92, 95%CI: 0.86–0.99), respectively. In the students of the engineering and business faculty, the frequency of presenting greater anxiety was only maintained at 11% (PR = 1.11, 95%CI: 1.00–1.22). Likewise, the association was maintained to present greater anxiety, depression and stress in those students who worked in a different health area or did not work, and those who were not diagnosed with COVID-19.

## 4. Discussion

Although previous research has been carried out on the impact on the mental health of university students during the pandemic [7,8], most have been conducted during the first months of the pandemic and have focused on evaluating depression or anxiety and their associated factors [9,10], without considering academic and clinical variables, which our study attempted to evaluate. Additionally, other studies have focused specifically on medical students and have not evaluated the impact of COVID-19 on the mental health of students in other majors [11,12]. Our research is relevant because it considered students from different faculties and delved into sociodemographic, academic, and clinical variables.

### 4.1. Prevalence of Mental Health Disorders

In the present investigation we found that 19.2% of the students presented depression. This is similar to the results of the study by Yusvisaret et al. (2021), who reported that 17% of students had depression in a private university in Mexico [15]. However, it differs from the result reported by Huarcaya-Victoria et al. (2021) who in their study of Peruvian university students reported levels of up to 74% of depression among students [16]. The prevalence found could be explained because our study was carried out in a city that presented high mortality from COVID-19 (approximately fifty thousand deaths by that date), which is a potential reason for the depression incidence [17]. Another possible explanation is due to periods of social distancing and isolation, which could influence the appearance of symptoms of depression [18]. Finally, the use of other scales (Zung, PHQ-9), may have provided different results.

We found that 23.2% of students presented anxiety. The findings correlate with the study by Chávez-Márquez (2021) who found a 30% prevalence of anxiety in students at a university in Mexico during the COVID-19 pandemic [19]. However, our result is lower than that reported by Saravia-Bartra et al. (2020), who in their study of medical students in Peru found that 75.4% manifested some degree of anxiety [20]. It is worth noting that this study was conducted during the first half of 2020, months in which the pandemic had just begun and the highest mortality rates in the country were recorded; while our study was carried out during the last months of the year 2020 when the contagion numbers had decreased, and vaccines were on the horizon. Likewise, the anxiety figure found in our research could also be explained due to the use of a different scale than the previous works (i.e., the GAD-7 scale) and the virtuality of the education process, which meant a process of change and adaptation for the students [21].

17.2% of the students surveyed presented stress. This is similar to the findings of Virto-Farfan et al. (2021) who reported a prevalence of stress in 15% of university students in Peru [22]. However, this result is lower than that documented by Luque-Vilca et al. (2022) who found that 92% of university students presented academic stress during the semester of study [23]. This is explained due to the various stress factors that intervene in the health of students, especially the overload of assignments and projects, which students must submit by a set deadline. Added to this is the abrupt change to on-line learning, which represents an additional stress for students due to the adaptation to this new modality.

### 4.2. Factors Associated with Mental Health Disorders

Women had a lower frequency of depression, anxiety, and stress. Although various studies show that women have a higher prevalence of depression, anxiety, and stress than men [24,25], the data regarding university students in the context of COVID-19 are not conclusive [26]. Our results differ from those reported by Soto-Rodriguez et al. (2021) who in their study showed that women had a higher prevalence of symptoms such as depression and anxiety during the pandemic [27]. This sex-related association could be explained by biological, hormonal, and psychosocial factors which explain the higher prevalence of these symptoms compared to men [28].

Likewise, the students of the engineering and business faculty had a higher frequency of anxiety. The findings correlate with the study by Siddiqui et al. (2020) who found that engineering students had a higher prevalence of symptoms of anxiety and depression compared to medical students [29]. However, it differs with various studies that conclude that medical students experience greater anxiety, unlike other careers [30,31]. Although it has been shown that health sciences students are more educated regarding viral pandemics and health concerns than other careers [32], which could reduce symptoms of anxiety and depression [33,34], the COVID pandemic has affected to students of all careers alike, due to the suspension of classes and face-to-face practices, social isolation and adaptation to the virtual education and learning process [35]. An additional explanation for this result may be due to variables such as preconceptions, fear or lived experiences rather than the educational career; however, these variables were not measured in our study.

Students who worked in an area other than health or did not work presented a higher frequency of depression, anxiety and stress compared to those who worked in a health-related area. This result differs from most research which focuses on health care personnel and finds a high prevalence of these three symptoms [36,37], which is explained by the high level of physical and psychological exhaustion due to work overload during the COVID-19 pandemic as the first line of response and by the latent risk of contagion [38,39]. However, most of these studies were conducted during the first months of the pandemic, when the number of deaths was high; in addition, the present investigation did not measure whether those who worked in a health-related area were necessarily on the front line attending COVID-19 cases.

In our study, we found that students who lived alone had a 27% lower frequency of anxiety compared to those who lived with others. This finding is similar to the study by Fingerman et al. (2021), who found that living alone was associated with positive emotions, which is explained by a decreased fear of infecting family and friends [40]. However, it differs from that reported by Marmet et al. (2021) & Raj et al. (2021), who found in their studies symptoms of psychological trauma, anxiety, depression, and decreased sleep in those people who lived alone during the pandemic period. This is consisted with the negative impact of isolation on mental health and by the perceived financial vulnerability of the study population [41,42].

Students who had been diagnosed with COVID-19 had a higher frequency of depression, anxiety, and stress. This finding correlates with the studies by Sher (2021) and Deng et al. (2020), who in their research found a high prevalence of symptoms related to depression and anxiety, which is likely the result of the stress experienced during the period of infection and by the long-term complications that may be triggered [43,44]. However, recent studies report a lower frequency of these symptoms in patients who had COVID-19, and this is possibly the product of two main factors; firstly, knowledge about the disease and secondly, a decreased risk perception resulting from the vaccination campaign [45,46]. Interestingly, at the time of execution of our study, the vaccination process had not yet started; yet, a growing body of knowledge of vaccines to combat COVID-19 was available and could have lessened some anxiety as well. This assumption should be addressed in detail measuring questions on vaccination and how this influenced on mental status.

There are other factors that may influence the development of mental disorders in university students. For example, further aspects such as stress level of studies [1], delay of studies due to the pandemic [2], or stressful thoughts regarding further waves of the pandemic [3]. These conditions may have increased the risk of mental disorders in some individuals compared to others. For this reason, it would be necessary to approach how the pandemic have affected long-term outcomes in education, such as academic performance and student burnout.

### 4.3. Limitations and Strenghts

The results of our study need to be placed in proper context and cautiously extrapolated and analyzed accordingly as there are both strengths and weaknesses in the data. First, as it is a cross-sectional study, causal relationships between the variables of interest evaluated cannot be affirmed. Second, the findings cannot be inferred to the entire population of university students, since they correspond to an analysis of only one university campus; therefore, selection bias could exist and, in addition, the population of this study was heterogeneous; consequently, extrapolations of the results should be carefully analyzed. Third, it was not possible to evaluate other variables that influence the development of mental health disorders in university students such as alcohol use, drug use, previous traumas or mental disorders, use of antidepressants and anti-anxiety drugs, income level, etc., which could lead to probable measurement bias. However, the main strength lies in the use of the DASS-21 instrument, which has adequate psychometric properties. This scale has been validated in Peru for multiple studies in university students [13,14], and, although it is not designed for diagnostic purposes, its use may help to understand how many students may potentially develop mental disorders. Another strength is that we included university students not only from health areas, which provides solid evidence on the impact of the COVID-19 pandemic on the mental health of university students from many different disciplines.

## 5. Conclusions

The association to present depression, anxiety and stress was lower in female students, while this frequency was higher in students who worked in a different area of health or did not work, and those who were not diagnosed with COVID-19. The students of the engineering and business faculty only had a higher frequency to present anxiety, but not depression and stress. Our results suggest the importance of promoting mental health awareness campaigns in university students due to the constant academic load they have.

## Figures and Tables

**Table 1:** Sociodemographic, academic, and clinical characteristics of the students.

Characteristics	N (%)
Sex	
Male	165 (41.2)
Female	235 (58.8)
Age *	24.5 ± 3.98
Marital status	
Non united (single, widowed, divorced)	378 (94.5)
United (married, cohabiting)	22 (5.5)
Faculty	
Health sciences	292 (73.0)
Engineering and business	85 (21.2)
Law and political science	23 (5.8)
Previous career	
Yes	93 (23.2)
No	307 (76.8)
Academic Year	
General studies (1st and 2nd year)	98 (24.5)
Faculty studies (3rd year or more)	302 (75.5)
Type of work	
Yes, health related	48 (12.0)
Yes, other than health	115 (28.8)
Does not work	237 (59.2)
With whom do you live?	
Accompanied	374 (93.5)
Alone	26 (6.5)
Do you have children?	
Yes	30 (7.5)
No	370 (92.5)
Have you been diagnosed with COVID-19?	
Yes	99 (24.8)
No	301 (75.2)
Has any family member been diagnosed with COVID-19?	
Yes	265 (66.2)
No	135 (33.8)
Depression **	
Yes	77 (19.2)
No	323 (80.8)
Anxiety **	
Yes	93 (23.2)
No	307 (76.8)
Stress **	
Yes	69 (17.2)
No	331 (82.8)

* Mean ± standard deviation; ** Obtained by DASS-21.

**Table 2:** Factors associated with presenting depression, anxiety, and stress in students of the Universidad Norbert Wiener. Bivariate analysis.

Variables	Depression		*p* **	Anxiety		*p* **	Stress		*p* **
	Yes (*n* = 77)	No (*n* = 323)		Yes (*n* = 93)	No (*n* = 307)		Yes (*n* = 69)	No (*n* = 331)	
	*n* (%)	*n* (%)		*n* (%)	*n* (%)		*n* (%)	*n* (%)	
**Sex**			**<0.001**			**<0.001**			**<0.001**
Male	15 (3.7)	150 (37.5)		21 (5.3)	144 (36.0)		12 (3.0)	153 (38.3)	
Female	62 (15.5)	173 (43.3)		72 (18.0)	163 (40.8)		57 (14.3)	178 (44.5)	
**Age**	23.1 ± 3.1	24.8 ± 4.0	0.996	23.5 ± 3.6	24.8 ± 4.0	0.997	23.4 ± 3.63	24.7 ± 4.0	0.994
**Marital status**			0.214			0.563			0.297
Non united	75 (18.8)	303 (75.8)		89 (22.3)	289 (72.3)		67 (16.8)	311 (77.8)	
United	2 (0.5)	20 (5.0)		4 (1.0)	18 (4.5)		2 (0.5)	20 (5.0)	
**Faculty**			0.332			**0.002**			**0.016**
Health sciences	65 (16.3)	227 (56.8)		81 (20.3)	211 (52.8)		60 (15.0)	232 (58.0)	
Engineering and business	9 (2.3)	76 (19.0)		9 (2.3)	76 (19.0)		7 (1.8)	78 (19.5)	
Law and political science	3 (0.8)	20 (5.0)		3 (0.8)	20 (5.0)		2 (0.5)	21 (5.3)	
**Previous career**			0.529			0.220			0.354
Yes	20 (5.0)	73 (18.3)		26 (6.5)	67 (16.8)		19 (4.8)	74 (18.5)	
No	57 (14.3)	250 (62.5)		67 (16.8)	240 (60.0)		50 (12.5)	257 (64.3)	
**Academic Year**			0.355			0.953			
General studies (1st and 2nd year)	22 (5.5)	76 (19.0)		23 (5.8)	75 (18.8)		17 (4.3)	81 (20.3)	0.977
Faculty studies (3rd year or more)	55 (13.8)	247 (61.8)		70 (17.5)	232 (58.0)		52 (13.0)	250 (62.5)	
**Type of work**			**<0.001**			**<0.001**			**<0.001**
Yes, health related	22 (5.5)	26 (6.5)		24 (6.0)	24 (6.0)		22 (5.5)	26 (6.5)	
Yes, other than health	11 (2.8)	104 (26.0)		12 (3.0)	103 (25.8)		8 (2.0)	107 (26.8)	
Does not work	44 (11.0)	193 (48.3)		57 (14.3)	180 (45.0)		39 (9.8)	198 (49.5)	
**With whom do you live?**			0.123			**0.004**			0.059
Accompanied	69 (17.3)	305 (76.2)		81 (20.3)	293 (73.3)		61 (15.3)	313 (78.3)	
Alone	8 (2.0)	18 (4.5)		12 (3.0)	14 (3.5)		8 (2.0)	18 (4.5)	
**Do you have children?**			0.393			0.991			0.274
Yes	4 (1.0)	26 (6.5)		7 (1.8)	23 (5.8)		3 (0.8)	27 (6.8)	
No	73 (18.3)	297 (74.3)		86 (21.5)	284 (71.0)		66 (16.5)	304 (76.0)	
**Have you been diagnosed with COVID-19?**			**<0.001**			**<0.001**			**<0.001**
Yes	42 (10.5)	57 (14.3)		46 (11.5)	53 (13.3)		39 (9.8)	60 (15.0)	
No	35 (8.8)	266 (66.5)		47 (11.8)	254 (63.5)		30 (7.5)	271 (67.8)	
**Has any family member been diagnosed with COVID-19?**			**<0.001**			**<0.001**			**0.001**
Sí	67 (16.8)	198 (49.5)		74 (18.5)	191 (47.8)		58 (14.5)	207 (51.8)	
No	10 (2.5)	125 (31.3)		19 (4.8)	116 (29.0)		11 (2.8)	124 (31.0)	

** *p*-values calculated with the Chi-Square test of independence.

**Table 3:** Factors associated with presenting depression, anxiety, and stress in students of the Universidad Norbert Wiener. Simple regression.

Characteristics	Simple Regression								
	Depression			Anxiety			Stress		
	PR	95%CI	*p* *	PR	95%CI	*p* *	PR	95%CI	*p* *
**Sex**									
Male	Ref			Ref			Ref		
Female	0.80	0.73–0.88	**<0.001**	0.79	0.71–0.88	**<0.001**	0.81	0.75–0.88	**<0.001**
**Age**	1.01	1.01–1.02	**<0.001**	1.01	1.00–1.02	**0.002**	1.01	1.00–1.02	**0.005**
**Marital status**									
Non united	Ref			Ref			Ref		
United	1.13	0.98–1.30	0.081	1.07	0.87–1.31	0.517	1.10	0.96–1.27	0.163
**Faculty**									
Health sciences	Ref			Ref			Ref		
Engineering and business	1.15	1.04–1.26	**0.004**	1.23	1.11–1.37	**<0.001**	1.15	1.05–1.25	**0.001**
Law and political science	1.11	0.94–1.32	0.196	1.20	1.01–1.43	**0.037**	1.14	0.99–1.32	0.050
**Previous career**									
Yes	Ref			Ref			Ref		
No	1.03	0.92–1.16	0.546	1.08	0.94–1.24	0.252	1.05	0.93–1.17	0.384
**Academic Year**									
General studies (1st and 2nd year)	Ref			Ref			Ref		
Faculty studies (3rd year or more)	1.05	0.93–1.18	0.382	1.00	0.88–1.13	0.953	1.00	0.90–1.11	0.977
**Type of work**									
Yes, health related	Ref			Ref			Ref		
Yes, other than health	1.66	1.27–2.18	**<0.001**	1.79	1.34–2.39	**<0.001**	1.71	1.31–2.23	**<0.001**
Does not work	1.50	1.15–1.96	**0.003**	1.51	1.13–2.03	**0.005**	1.54	1.18–2.01	**0.001**
**With whom do you live?**									
Accompanied	Ref			Ref			Ref		
Alone	0.81	0.77–0.85	0.219	0.68	0.47–0.98	**0.041**	0.82	0.63–1.07	0.153
**Do you have children?**									
Yes	Ref			Ref			Ref		
No	0.92	0.79–1.07	0.314	1.00	0.81–1.22	0.991	0.91	0.80–1.03	0.165
**Have you been diagnosed with COVID-19?**									
Yes	Ref			Ref			Ref		
No	1.53	1.28–1.82	**<0.001**	1.57	1.30–1.90	**<0.001**	1.48	1.26–1.74	**<0.001**
**Has any family member been diagnosed with COVID-19?**									
Yes	Ref			Ref			Ref		
No	1.23	1.13–1.34	**<0.001**	1.19	1.07–1.31	**0.001**	1.17	1.08–1.27	**<0.001**

* *p*-values obtained with Generalized Linear Models (GLM), Poisson family, log-link function and robust variance.

**Table 4:** Factors associated with depression, anxiety, and stress in students of the Universidad Norbert Wiener. Multiple regression.

Characteristics	Multiple Regression **								
	Depression			Anxiety			Stress		
	PR	95%CI	*p* *	PR	95%CI	*p* *	PR	95%CI	*p* *
**Sex**									
Male	Ref			Ref			Ref		
Female	0.91	0.84–0.99	**0.028**	0.90	0.83–0.99	**0.033**	0.92	0.86–0.99	**0.037**
**Age**	1.03	1.02–1.04	**<0.001**	1.03	1.01–1.05	**<0.001**	1.02	1.01–1.04	**<0.001**
**Faculty**									
Health sciences	Ref			Ref			Ref		
Engineering and business	1.04	0.95–1.13	0.313	1.11	1.00–1.22	**0.033**	1.04	0.97–1.12	0.204
Law and political science	1.02	0.89–1.17	0.713	1.07	0.92–1.24	0.345	1.03	0.94–1.14	0.456
**Type of work**									
Yes, health related	Ref			Ref			Ref		
Yes, other than health	1.37	1.07–1.78	**0.012**	1.39	1.06–1.82	**0.017**	1.39	1.08–1.78	**0.010**
Does not work	1.45	1.11–1.90	**0.006**	1.36	1.01–1.82	**0.039**	1.42	1.08–1.87	**0.011**
**With whom do you live?**									
Accompanied	Ref			Ref			Ref		
Alone	0.81	0.77–0.85	0.219	0.73	0.56–0.95	**0.020**	0.82	0.63–1.07	0.153
**Have you been diagnosed with COVID-19?**									
Yes	Ref			Ref			Ref		
No	1.20	1.03–1.39	**0.017**	1.19	1.01–1.40	**0.029**	1.20	1.04–1.38	**0.010**
**Has any family member been diagnosed with COVID-19?**									
Yes	Ref			Ref			Ref		
No	1.00	0.93–1.08	0.906	0.93	0.85–1.02	0.149	0.97	0.90–1.04	0.477

* *p*-values obtained with Generalized Linear Models (GLM). ** Adjusted for age, sex, type of work and COVID-19 diagnosis.

## Data Availability

The dataset generated and analyzed during the current study is not publicly available because the ethics committee has not provided permission/authorization to publicly share the data but are available from the corresponding author on reasonable request.
